# HRP-2 determines HIV-1 integration site selection in LEDGF/p75 depleted cells

**DOI:** 10.1186/1742-4690-9-84

**Published:** 2012-10-09

**Authors:** Rik Schrijvers, Sofie Vets, Jan De Rijck, Nirav Malani, Frederic D Bushman, Zeger Debyser, Rik Gijsbers

**Affiliations:** 1 Division of Molecular Medicine, Katholieke Universiteit Leuven, Leuven, Flanders, Belgium; 2 Department of Microbiology, University of Pennsylvania School of Medicine, Philadelphia, Pennsylvania, USA

**Keywords:** LEDGF/p75, HRP-2, HIV-1, Targeting, Integration site analysis

## Abstract

**Background:**

Lens epithelium–derived growth factor (LEDGF/p75) is a cellular co-factor of HIV-1 integrase (IN) that tethers the viral pre-integration complex to the host cell chromatin and determines the genome wide integration site distribution pattern of HIV-1. Recently, we demonstrated that HIV-1 replication was reduced in LEDGF/p75 knockout (KO) cells. LEDGF/p75 KO significantly altered the integration site preference of HIV-1, but the pattern remained distinct from a computationally generated matched random control set (MRC), suggesting the presence of an alternative tethering factor. We previously identified Hepatoma-derived growth factor related protein 2 (HRP-2) as a factor mediating LEDGF/p75-independent HIV-1 replication. However, the role of HRP-2 in HIV-1 integration site selection was not addressed.

**Findings:**

We studied the HIV-1 integration site distribution in the presence and absence of LEDGF/p75 and/or HRP-2, and in LEDGF/p75-depleted cells that overexpress HRP-2. We show that HRP-2 functions as a co-factor of HIV-1 IN in LEDGF/p75-depleted cells. Endogenous HRP-2 only weakly supported HIV-1 replication in LEDGF/p75 depleted cells. However, HRP-2 overexpression rescued HIV-1 replication and restored integration in RefSeq genes to wild-type levels. Additional HRP-2 KD in LEDGF/p75-depleted cells reduces integration frequency in transcription units and shifts the integration distribution towards random.

**Conclusions:**

We demonstrate that HRP-2 overexpression can compensate for the absence of LEDGF/p75 and indicate that the residual bias in integration targeting observed in the absence of LEDGF/p75 can be ascribed to HRP-2. Knockdown of HRP-2 upon LEDGF/p75 depletion results in a more random HIV-1 integration pattern. These data therefore reinforce the understanding that LEDGF/p75 is the dominant HIV-1 IN co-factor.

## Findings

**Figure 1:**
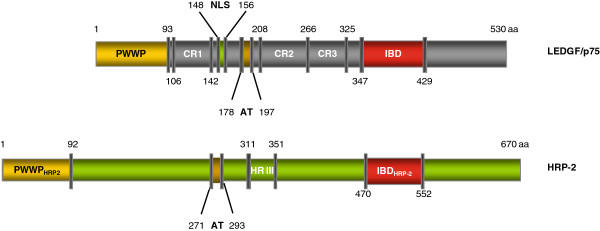
** Structure of LEDGF/p75 and HRP-2. ** Cartoon representation of LEDGF/p75 and HRP-2: PWWP-domain (PWWP), charged region 1–3 (CR1-3), nuclear localization signal (NLS), AT hook-like sequence (AT), homology region III (HR III) [9] and integrase binding domain (IBD).

HIV-1 integration target site selection is not a random event, but preferentially occurs in the body of active transcription units [1], due to the interaction with the cellular co-factor LEDGF/p75 [2,3]. Knockdown (KD) or KO of LEDGF/p75 shifts HIV-1 integration targeting away from transcription units [2-7]. LEDGF/p75 functions as a molecular tether, interacting with the viral integrase (IN) via its C-terminal integrase binding domain (IBD) [8,9], and with the host-cell chromatin via its N-terminus. The latter contains chromatin-binding motifs such as the PWWP-domain, AT-hook-like motifs, and a set of charged regions (Figure 1) [6,10]. Several lines of evidence support this tethering and targeting model for LEDGF/p75 in HIV-1 replication. The chromatin binding sites of LEDGF/p75 correlate with the HIV-1 integration distribution pattern [11], and LEDGF/p75-hybrids in which the N-terminus is replaced by an alternative chromatin interaction domain, such as the heterochromatin binding element CBX1, have been shown to retarget HIV-1 integration out of transcription units and towards heterochromatic regions [5,12]. 

We reported that LEDGF/p75 KO shifts HIV-1 integration away from transcription units [7], consistent with previous LEDGF/p75 KD experiments [2,3]. However, even in the absence of LEDGF/p75, HIV-1 significantly favored integration in transcription units when compared to random [7]. This may be explained by an intrinsic feature of the IN protein or the pre-integration complex, or by the presence of one or more additional co-factors that target integration into transcription units. Recently, we reported that HRP-2, the only known cellular protein besides LEDGF/p75 that combines a PWWP-domain and an IBD-like domain [9,13] (Figure 1), plays a role in LEDGF/p75-independent HIV-1 replication in both human LEDGF/p75 KO and KD cell lines [7]. In this study we examined the contribution of HRP-2 in directing integration site selection of HIV-1.

**Figure 2:**
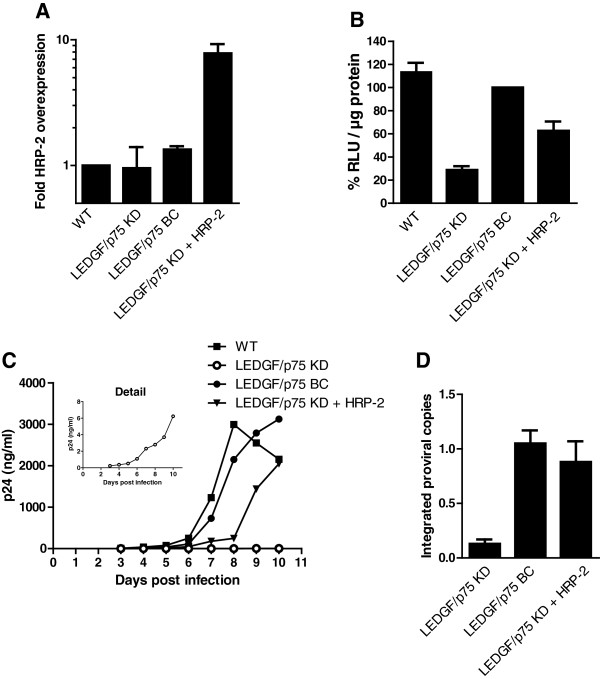
** HRP-2 overexpression rescues HIV-1 replication. ** WT and stable LEDGF/p75 KD cell lines (LEDGF/p75 KD) were complemented with LEDGF/p75 (LEDGF/p75 BC) or HRP-2 (LEDGF/p75 KD + HRP-2). (**A**) HRP-2 mRNA expression levels shown as fold overexpression compared to WT. (**B**) Relative luciferase activity (RLU/μg protein) following HIV-fLuc transduction. Data were compiled from at least six independent experiments and expressed as percentages relative to LEDGF/p75 BC (mean ± SD). (**C**) Multiple round HIV-1 replication after challenging the indicated cell lines with the laboratory strain HIV-1_NL4.3_. Replication was monitored by measuring the p24 content in the supernatant until day 10 at which point cells were confluent or showed massive cell death due to CPE. P24 levels decreased after day 8 in the WT condition due to CPE. Experiments were performed at least three times, a representative experiment is shown. (**D**) At day 10 of the experiment shown in (**C**), cells were split and maintained under antiretroviral therapy (AZT, 50xIC_50_) for 10 days to dilute all non-integrated viral DNA forms, before determining the number of integrated copies using qPCR.

We and others have demonstrated that back-complementation (BC) of LEDGF/p75 in LEDGF/p75-depleted cells rescued HIV-1 replication and restored the integration site distribution to wild-type (wild-type) patterns [5,14]. Here, we first assessed the potential of HRP-2 to complement LEDGF/p75-depleted cells. Overexpression of HRP-2 (9-fold compared to endogenous wild-type levels, Figure 2A) in LEDGF/p75-depleted cells could substitute in part for LEDGF/p75. Infection with single-round HIV-fLuc virus was restored to 62 ± 8% compared to LEDGF/p75 BC cells (Figure 2B, compare LEDGF/p75 BC and LEDGF/p75 KD + HRP-2). Replication of HIV-1_NL4.3_ virus was rescued to near wild-type levels upon HRP-2 overexpression as seen with LEDGF/p75 BC (Figure 2C). Both LEDGF/p75 and HRP-2 mediated rescue correlated with an increase in integrated proviral copies (Figure 2D). Next, in line with previous observations [15], the nuclear distribution pattern of LEDGF/p75 during interphase was speckled, while that of HRP-2 was homogenous (Additional file 1, compare row 2 and 3). Unlike LEDGF/p75, HRP-2 did not bind mitotic chromosomes (Additional file 1, compare row 5 and 6), although this might not be relevant for HIV-1 replication since LEDGF/p75 depletion in non-dividing macrophages also affects HIV-1 replication [16]. Still, HRP-2 overexpression relocated IN to the nucleus in LEDGF/p75 KD cells (Additional file 1, row 3), suggesting a direct interaction. 

Previously, we demonstrated that HRP-2 KD in LEDGF/p75-depleted cells additionally hampered HIV-1 replication (see reference [7] and data not shown). Here, we evaluated the effect of additional HRP-2 KD in LEDGF/p75-depleted cells on HIV-1 integration site distribution. We generated stable HRP-2 KD and LEDGF/p75 KD cells (86% and >90% KD on mRNA level for HRP-2 and LEDGF/p75, respectively), double KD cells (>90% LEDGF/p75 KD and 84% HRP-2 KD on mRNA level) and complemented LEDGF/p75 KD cells with HRP-2 (Figure 2A and data not shown). The respective cell lines were challenged with HIV-fLuc integration sites were amplified and their distribution pattern was characterized.

**Figure 3:**
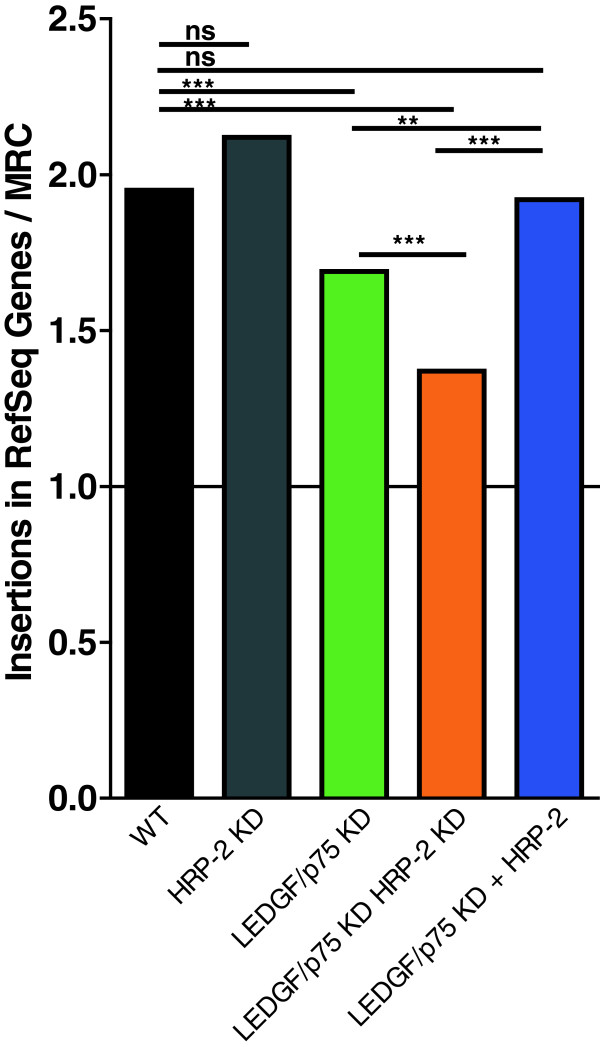
** HRP-2 affects HIV-1 integration in genes.** Integration events of HIV-1 (HIV-fLuc) in genes according to the RefSeq annotation were normalized to MRC and presented per cell line. The line at y = 1.0 represents random integration (equal to MRC). Asterisks represent statistical significance of differences between two data sets, indicated by the line above the two sets, with * p < 0.05, ** p < 0.01, *** p < 0.001, using a two-tailed Chi-Square test. Statistical methods are described in detail by Berry *et al.*[20]. Analysis was carried out using The R Project for Statistical Computing software. P-values were not corrected for multiple comparisons; *alpha* level is 0.007 after Bonferroni correction (0.05/7).

**Figure 4:**
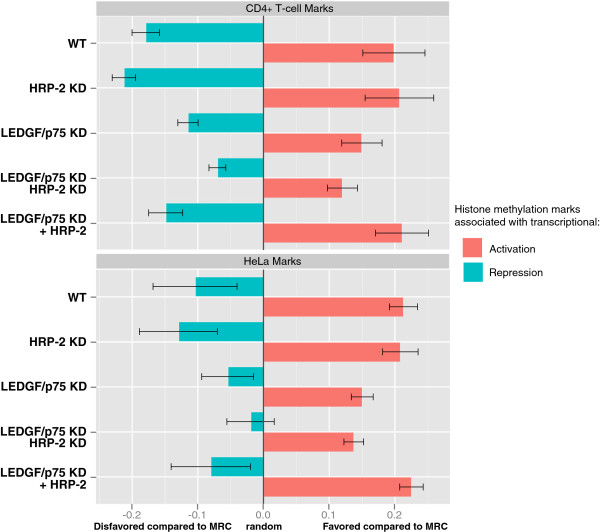
** LEDGF/p75 and HRP-2 depletion shifts integration towards random.** A pooled score was determined for HIV-1 integration near epigenetic marks associated with transcriptional repression or activation for each cell line and plotted in a bar diagram. The X-axis represents deviation from random with positive values indicating favored integration compared to MRC, negative values disfavored integration compared to MRC. The pooled score is the average of the different ROC values - 0.5 (to indicate the deviation from random) obtained with individual markers (not shown) in a 10 kb window. For HeLaP4, H3K9me2, H3K9me3, H3K27me3, and H4K20me3 were pooled as associated with ‘*repression*’; H2BK5me1, H3K4me1, H3K36me3, H4K20me1 were pooled as associated with ‘*activation*’. For CD4^+^ T-cells, H3K9me2, H3K9me3, H3K27me2, H3K27me3, H3K79me3 and H4K20me3 were pooled as associated with ‘*repression*’; H2BK5me1, H3K4me1, H3K4me2, H3K9me1, H3K27me1, H3K36me3, H4K20me1 were pooled as associated with ‘*activation*’.

First, we generated a genomic heat map describing the integration site distribution for a subset of genomic features (Additional file 2). The color of each tile represents the deviation from random (MRC = 0.5) for the examined feature, ranging from red (favored compared to MRC) over white to blue (disfavored compared to MRC). While tile colors did not differ when HRP-2 was depleted in wild-type cells (compare wild-type and HRP-2 KD), a shift in tile color towards random could be appreciated upon LEDGF/p75 KD (compare wild-type and LEDGF/p75 KD), which was even more pronounced upon the additional suppression of HRP-2 expression (compare LEDGF/p75 KD and LEDGF/p75 KD HRP-2 KD). More detailed analysis showed that integration in RefSeq genes was favored in wild-type cells (77.5% in genes, p < 0.001 compared to MRC) and decreased significantly upon LEDGF/p75 KD (67.7% in genes, p < 0.001 compared to MRC), consistent with previous results [2,5,6] (Table 1, Figure 3). HRP-2 overexpression in LEDGF/p75 KD cells rescued proviral integration in transcription units (77.5%, no difference compared to wild-type; Table 1, Figure 3 and Additional file 2). KD of HRP-2 in wild-type cells did not affect integration site distribution (80.2% in genes, p = 0.3 for the comparison of wild-type and HRP-2 KD), confirming the dominant role of LEDGF/p75 over HRP-2. However, KD of HRP-2 in LEDGF/p75-depleted cells resulted in an additional decrease of integration in RefSeq genes, shifting integration out of transcription units and towards random (67.7% in LEDGF/p75 KD *versus* 53.7% in LEDGF/p75 KD HRP-2 KD, p < 0.001) (Table 1, Figure 3). Together these data provide evidence for a role of HRP-2 in targeting HIV-1 integration in LEDGF/p75-depleted cells. Using a panel of histone modifications we also evaluated the frequency of integration near transcriptionally repressed regions (either silent regions, e.g. H3K27me3, or heterochromatin, e.g. H3K9me3, H3K79me3, H4K20me3), as well as near marks associated with activation (e.g. H2BK5me1, H3K9me1, and H4K20me1). Since only a limited number of marks have been mapped in HeLa cells [17], we also included marks that were defined in CD4^+^ T-cells [18] (Figure 4). HIV-1 integration in wild-type cells is generally disfavored near marks associated with transcriptionally silent regions and heterochromatin. LEDGF/p75 depletion shifts this phenotype more to MRC. In line with our previous data, additional HRP-2 KD in LEDGF/p75-depleted cells shifted integration distribution more towards MRC, indicating that the integration is more random upon double KD (Figure 4, compare LEDGF/p75 KD and LEDGF/p75 KD HRP-2 KD). Likewise, integration near histone marks associated with active transcription is reduced upon LEDGF/p75 KD and even more upon additional HRP-2 KD. HRP-2 overexpression restored the integration pattern near histone marks to levels observed in wild-type cells. The possible effects of such altered integration site distribution on proviral gene expression remain to be investigated. Since LEDGINs [19], allosteric IN inhibitors targeting the LEDGF/p75 binding site in IN, interfere with the interaction of either LEDGF/p75 or HRP-2 [7], LEDGINs might affect HIV-1 integration site distribution. Comparable data were obtained when transducing the same cell lines with an HIV-derived lentiviral vector (Additional file 3; Table S1). 

**Table 1:** Integration frequency of HIV in RefSeq genes

	**Cell line**	**# sites**	**% in RefSeq genes**
**HIV-fLuc**	WT	1468	77.5 ^***|ns|***^
**sites**	HRP-2 KD	359	80.2 ^***|ns|***^
	LEDGF/p75 KD	477	67.7 ^***|***|ns^
	LEDGF/p75 KD HRP-2 KD	676	53.7 ^***|***|***^
	LEDGF/p75 KD + HRP-2	445	77.5 ^***|ns|***^
**MRC sites**	WT	4404	39.7
**(HIV-fLuc)**	HRP-2 KD	1077	37.9
	LEDGF/p75 KD	1431	40.3
	LEDGF/p75 KD HRP-2 KD	2028	39.3
	LEDGF/p75 KD + HRP-2	1335	40.2

*Abbreviations: MRC* matched random control, *ns* non-significant (p ≥ 0.05). Asterisks represent p-values (*p < 0.05, **p < 0.01, ***p < 0.001) given after comparison with MRC|WT|LEDGF/p75 KD respectively. Significance was determined using a two-tailed Chi-square test. P-values were not corrected for multiple comparisons; *alpha* level is 0.003 after Bonferroni-correction (0.05/15).

Taken together, our work underscores the dominant role of LEDGF/p75 over HRP-2 in HIV-1 replication, which can be explained by the lower affinity of HRP-2 for HIV-1 IN compared to LEDGF/p75 [7,9,15]. Since HRP-2 overexpression in LEDGF/p75-depleted cells rescues HIV-1 replication, the relative expression levels of LEDGF/p75 and HRP-2 in relevant primary host cells are of importance. Different groups have reported on expression levels of LEDGF/p75 in primary activated and resting T-cells [21] or in different patient populations [22,23], yet the levels in other subsets of HIV target-cells as well as the HRP-2 expression levels remain to be determined. Currently, there is no evidence that HRP-2 plays a role in HIV-1 replication in cells expressing WT levels of LEDGF/p75. Both LEDGF/p75 and HRP-2 carry a PWWP domain, recently identified as a chromatin reader recognizing epigenetic marks, such as methylated histone side-chains [6,24,25]. Previously, we demonstrated that swapping the PWWP-domain of LEDGF/p75 with that of HRP-2 could rescue lentiviral replication and integration site selection in genes [6]. Here, integration distribution in LEDGF/p75 KD cells overexpressing HRP-2 was comparable to wild-type cells (Figure 3 and Additional file 2 and 3), suggesting only subtle differences between LEDGF/p75 and HRP-2 for the interaction with the local chromatin. Although LEDGF/p75 and HRP-2 double KD shifted integration significantly out of transcription units, integration remained distinct from random in the double KD cells (Table 1). Multiple (mutually non-exclusive) reasons can be put forward to explain this observation. First, in these experiments we employed RNAi and even though the knockdown was potent (>90% and 84% on mRNA level for LEDGF/p75 and HRP-2 respectively), residual LEDGF/p75 and HRP-2 might account for the residual bias. The generation of a human double KO cell line, devoid of both LEDGF/p75 and HRP-2, will provide a more definite answer. Second, this result could suggest the presence of additional cellular co-factor(s) that affect targeting in the absence of LEDGF/p75 and HRP-2. Transcription factor IIS (TFIIS) for example harbors an IBD-like domain [6,8], although structurally more distantly related to the IBD of LEDGF/p75 and HRP-2, but lacks a PWWP-domain. However, in the presence of LEDGF/p75, HRP-2 does not seem to play a role in HIV replication [6] or targeting (this work), suggesting alternate IBD containing tethers will probably only play a minor role in HIV replication or targeting in WT conditions, unless expression levels differ strongly, accrediting LEDGF/p75 as principal tether [6]. Third, the bias might reflect specific constraints of the viral IN, the local chromatin environment or the pre-integration complex itself. Although HIV integration favors weak palindromic sequences, our analysis indicated that this preference is irrespective of the presence or absence of LEDGF/p75 and/or HRP-2 ( [6] and data not shown).

In conclusion, our data provide an explanation for why LEDGF/p75 depletion alone does not completely retarget integration distribution towards random, and they fit with previous data that HRP-2 binds the IN dimer with lower affinity [7,9]. These data also reinforce our understanding that LEDGF/p75 is the dominant cellular co-factor determining HIV-1 integration site selection.

## Competing interests

The authors declare no conflict of interest.

## Authors’ contributions

RS, SV, JDR, ZD, and RG conceived and designed the experiments. RS, SV, JDR, RG performed the experiments. RS, NM, FDB, ZD, RG analyzed the data. RS, RG wrote the paper. All authors read and approved the final manuscript.

## Funding

RS and JDR are doctoral fellows of the Flemish Fund for Scientific Research (FWO Vlaanderen), SV is a doctoral fellow of the Iwild-type. JDR is holder of a Mathilde-Krim postdoctoral fellowship (amfAR). Research was funded by grants from the Iwild-type (SBO grant CellCoVir), the FWO and the EU (FP7 THINC). This work was supported by NIH grants AI52845 and AI082020, the University of Pennsylvania Center for AIDS Research, and the Penn Genome Frontiers Institute with a grant with the Pennsylvania Department of Health. The funders had no role in study design, data collection and analysis, decision to publish, or preparation of the manuscript.

## Supplementary Material

Additional file 1 ** HRP-2 overexpression relocates integrase to the nucleus of LEDGF/p75 depleted cells.** Cells were transfected with plasmid encoding mRFP-IN and laser scanning microscopy images of cells stained with anti-LEDGF/p75 (LEDGF/p75) or anti-Flag (HRP-2) antibody are shown (green). Nuclei were stained with DAPI (4’,6-diamidino-2-phenylindole; blue). The respective constructs and cell lines are indicated. Interphase and mitotic cells are displayed separately. The data are representative for the vast majority of the imaged cells. Click here for file

Additional file 2 ** Heat map of integration frequency relative to genomic features.** Heat maps were developed to summarize relationships of proviral integration sites with genomic features using the receiver operating characteristic (ROC) area method [20]. The analyzed genomic features are mentioned on the left of the corresponding row of the heat map. Tile color indicates whether a chosen feature is favored (*red*, enrichment compared with random) or disfavored (*blue*, depletion compared with random) for integration for the respective data sets relative to their MRCs, as detailed in the colored ROC area scale at the *bottom of the panel*. The different data sets used are indicated above the columns. The *asterisks* denote significant differences of HIV integration compared to the LEDGF/p75 KD cell line for the respective features (*, *p* < 0.05, **, p < 0.01; ***, *p* < 0.001, using Wald statistics referred to a Chi-square distribution), *dashes* overlay control tiles. The naming of the genomic features is described in Berry *et al.*[20]; *TSS*, transcription start site. Click here for file

Additional file 3 ** Table S1. ** Integration frequency of HIV-derived lentiviral vector in RefSeq genes. Click here for file
