# Supplementary figures and images for: HRP-2 determines HIV-1 integration site selection in LEDGF/p75 depleted cells
Source: Retrovirology. 2012 Oct 9;9:84. doi: 10.1186/1742-4690-9-84 (PMC3485173; doi:10.1186/1742-4690-9-84)

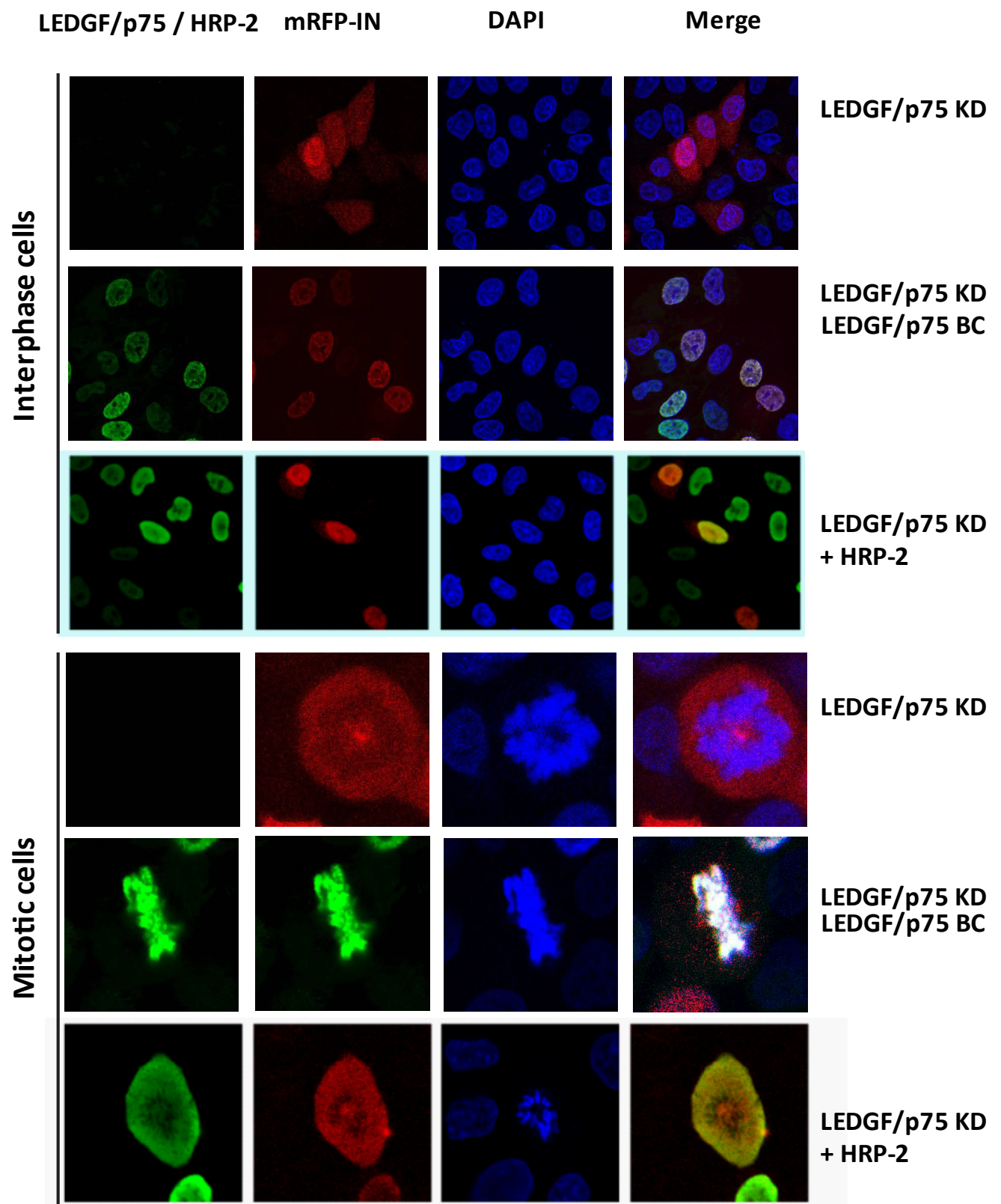

Supplement: Additional file 1 — HRP-2 overexpression relocates integrase to the nucleus of LEDGF/p75 depleted cells. Cells were transfected with plasmid encoding mRFP-IN and laser scanning microscopy images of cells stained with anti-LEDGF/p75 (LEDGF/p75) or anti-Flag (HRP-2) antibody are shown (green). Nuclei were stained with DAPI (4’,6-diamidino-2-phenylindole; blue). The respective constructs and cell lines are indicated. Interphase and mitotic cells are displayed separately. The data are representative for the vast majority of the imaged cells. [file 1742-4690-9-84-S1.pdf]

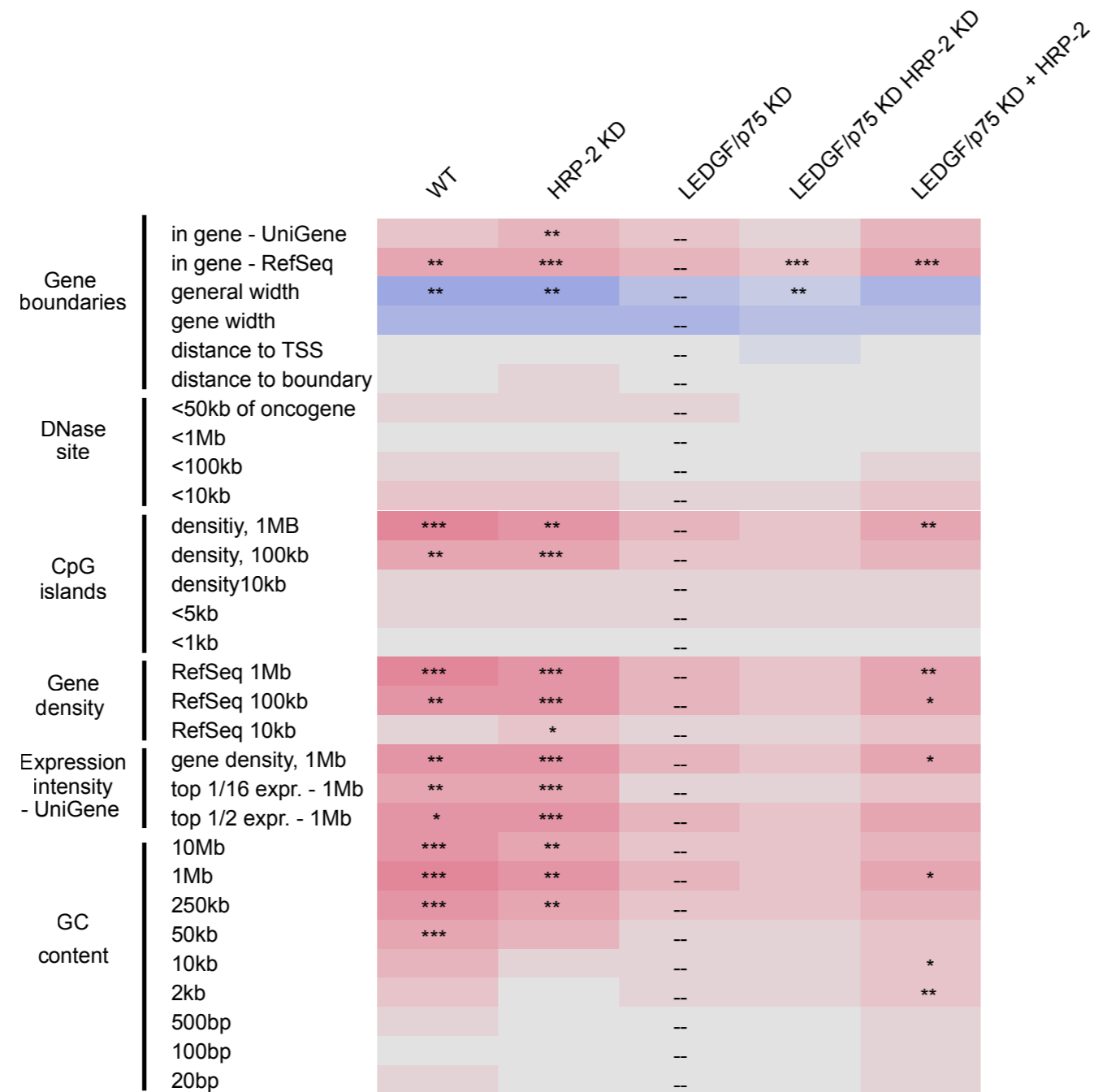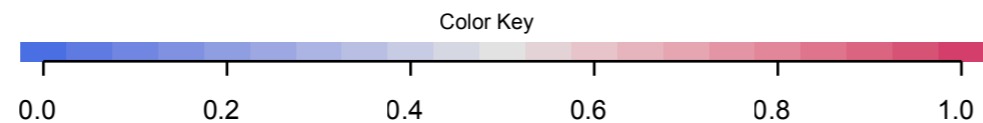

Supplement: Additional file 2 — Heat map of integration frequency relative to genomic features. Heat maps were developed to summarize relationships of proviral integration sites with genomic features using the receiver operating characteristic (ROC) area method [20]. The analyzed genomic features are mentioned on the left of the corresponding row of the heat map. Tile color indicates whether a chosen feature is favored (red, enrichment compared with random) or disfavored (blue, depletion compared with random) for integration for the respective data sets relative to their MRCs, as detailed in the colored ROC area scale at the bottom of the panel. The different data sets used are indicated above the columns. The asterisks denote significant differences of HIV integration compared to the LEDGF/p75 KD cell line for the respective features (*, p < 0.05, **, p < 0.01; ***, p < 0.001, using Wald statistics referred to a Chi-square distribution), dashes overlay control tiles. The naming of the genomic features is described in Berry et al.[20]; TSS, transcription start site. [file 1742-4690-9-84-S2.pdf]
