# Supplementary material for: HRP-2 determines HIV-1 integration site selection in LEDGF/p75 depleted cells
Source: Retrovirology. 2012 Oct 9;9:84. doi: 10.1186/1742-4690-9-84 (PMC3485173; doi:10.1186/1742-4690-9-84)
Supplement: Additional file 3 — Table S1. Integration frequency of HIV-derived lentiviral vector in RefSeq genes. [file 1742-4690-9-84-S3.pdf]

**Table S1. Integration frequency of HIV-derived lentiviral vector in RefSeq genes**

| | **Cell line** | **# sites** | **% in RefSeq genes** |
|---|---|---|---|
| **LV sites** | WT | 1174 | $73.3^{***\vert ns\vert ***}$ |
| | HRP-2 KD | 2153 | $69.7^{***\vert *\vert ***}$ |
| | LEDGF/p75 KD | 880 | $57.3^{***\vert ***\vert ns}$ |
| | LEDGF/p75 KD HRP-2 KD | 4480 | $52.4^{***\vert ***\vert **}$ |
| | LEDGF/p75 KD + HRP-2 | 1553 | $65.7^{***\vert ***\vert ***}$ |
| **MRC sites (LV)** | WT | 3522 | 39.2 |
| | HRP-2 KD | 6459 | 40.2 |
| | LEDGF/p75 KD | 2640 | 38.9 |
| | LEDGF/p75 KD HRP-2 KD | 13440 | 39.3 |
| | LEDGF/p75 KD + HRP-2 | 4659 | 40.9 |

*Abbreviations:* LV, lentiviral vector; MRC, matched random control; ns, non-significant ($p \geq 0.05$); asterisks represent p-values (*$p<0.05$, **$p<0.01$, ***$p<0.001$) given after comparison with MRC|WT|LEDGF KD respectively. Significance was determined using a two-tailed Chi-square test. P-values were not corrected for multiple comparisons; *alpha* level is 0.003 after Bonferroni-correction (0.05/15).
